# Development and Validation of Analytical Method for SH-1242 in the Rat and Mouse Plasma by Liquid Chromatography/Tandem Mass Spectrometry

**DOI:** 10.3390/molecules25030531

**Published:** 2020-01-25

**Authors:** Yoo-Seong Jeong, Minjeong Baek, Seungbeom Lee, Min-Soo Kim, Han-Joo Maeng, Jong-Hwa Lee, Young-Ger Suh, Suk-Jae Chung

**Affiliations:** 1 College of Pharmacy and Research Institute of Pharmaceutical Sciences, Seoul National University, 1 Gwanak-ro, Gwanak-gu, Seoul 08826, Korea; jus2401@snu.ac.kr (Y.-S.J.); jormungand@snu.ac.kr (M.B.); lastchaos21c@snu.ac.kr (S.L.); misol@snu.ac.kr (M.-S.K.); ygsuh@cha.ac.kr (Y.-G.S.); 2 College of Pharmacy, CHA University, 120 Haeryong-ro, Pocheon-si, Gyeonggi-do 11160, Korea; 3 College of Pharmacy, Gachon University, 191 Hambakmoei-ro, Yeonsu-gu, Incheon 21936, Korea; hjmaeng@gachon.ac.kr; 4 Korea Institute of Toxicology, 141 Gajeong-ro, Yuseong-gu, Daejeon 34114, Korea; jhl@kitox.re.kr

**Keywords:** SH-1242, 2-(3,4-dimethoxyphenyl)-1-(5-methoxy-2,2-dimethyl-2*H*-chromen-6-yl)ethanone, pharmacokinetics, HPLC-MS/MS

## Abstract

SH-1242, a novel inhibitor of heat shock protein 90 (HSP90), is a synthetic analog of deguelin: It was previously reported that the treatment of SH-1242 led to a strong suppression of hypoxia-mediated retinal neovascularization and vascular leakage in diabetic retinas by inhibiting the hypoxia-induced upregulation of expression in hypoxia-inducible factor 1α (HIF-1α) and vascular endothelial growth factor (VEGF). In this study, an analytical method for the quantification of SH-1242 in biological samples from rats and mice was developed/validated for application in pharmacokinetic studies. SH-1242 and deguelin, an internal standard of the assay, in plasma samples from the rodents were extracted with methanol containing 0.1% formic acid and analyzed at *m*/*z* transition values of 368.9→151.0 and 395.0→213.0, respectively. The method was validated in terms of accuracy, precision, dilution, matrix effects, recovery, and stability and shown to comply with validation guidelines when it was used in the concentration ranges of 1–1000 ng/mL for rat plasma and of 2–1000 ng/mL for mouse plasma. SH-1242 levels in plasma samples were readily determined using the developed method for up to 480 min after the intravenous administration of 0.1 mg/kg SH-1242 to rats and for up to 120 min to mice. These findings suggested that the current method was practical and reliable for pharmacokinetic studies on SH-1242 in preclinical animal species.

## 1. Introduction

Heat shock protein 90 (HSP90) is a chaperone protein that plays a vital role in the regulation of its target proteins via stabilizing them under cellular stresses, post-translational folding, and degrading damaged proteins [1]. Hypoxia-inducible factor 1 (HIF-1), one of the client proteins of HSP90, is a transcription factor that is associated with the promotion of angiogenesis in regions of vascular dysfunction, e.g., hypoxic cancer environments. HIF-1 has also been reported to facilitate the expression of vascular endothelial growth factor (VEGF) [2,3] and to be pathologically related to diabetic retinopathy and age-related macular degeneration, as well as cancers [4,5]. Deguelin is a naturally occurring rotenoid, a flavonoid, and has been shown to inhibit HSP90’s function [6]. Although its underlying mechanism has not been fully delineated, this inhibition appears to be related to the attenuation of the binding of clients to ATP-binding pocket of HSP90 by deguelin and to the accelerated decomposition of HIF-1 [6]. Previously, a variety of deguelin analogs were synthesized and screened for HSP90 inhibition [7,8]. In particular, we noted that SH-1242 (2-(3,4-dimethoxyphenyl)-1-(5-methoxy-2,2-dimethyl-2H-chromen-6-yl)ethanone) possessed a potent anti-proliferative activity against human cancer cells [9] and suppressed hypoxia-mediated retinal neovascularization/vascular leakage in diabetic retinas [10]. Furthermore, potent antitumor effects of SH-1242 were also found in various cancer cell lines and in vivo animal models with significantly reduced neurotoxicity [11], suggesting SH-1242 is a reasonable candidate as an experimental inhibitor of HSP90.

Since SH-1242 was found to exhibit a strong antiangiogenic activity in the low nM range (e.g., an effective concentration of approximately down to 10 nM in in vitro model of hypoxia-mediated angiogenesis) [10], a sensitive/robust quantification method for SH-1242, especially in complex biological matrices (e.g., plasma samples), is required for the further development of SH-1242 as a new drug. However, previous attempts to develop methodologies for the analyses of deguelin analogs have primarily focused on the determination of rotenoid levels in natural sources [12,13,14,15]. In particular, an analytical method capable of quantifying deguelin analogs in the nano-molar range in biological matrices from animal species used for preclinical study settings was not reported. Therefore, the objective of this study was to develop and validate an analytical method for the determination of SH-1242 in plasma samples of rats and mice in accordance with the U.S. Food and Drug Administration (US FDA) guidance [16]. We were particularly interested in developing the assay capable of measuring the rotenoid analogs at the lower limit of quantification (LLOQ) down to low ng/mL range for the application of the devised method to pharmacokinetic studies in the preclinical animal species.

## 2. Results and Discussion

### 2.1. Mass Spectrometry and Chromatography

**Figure 1:**
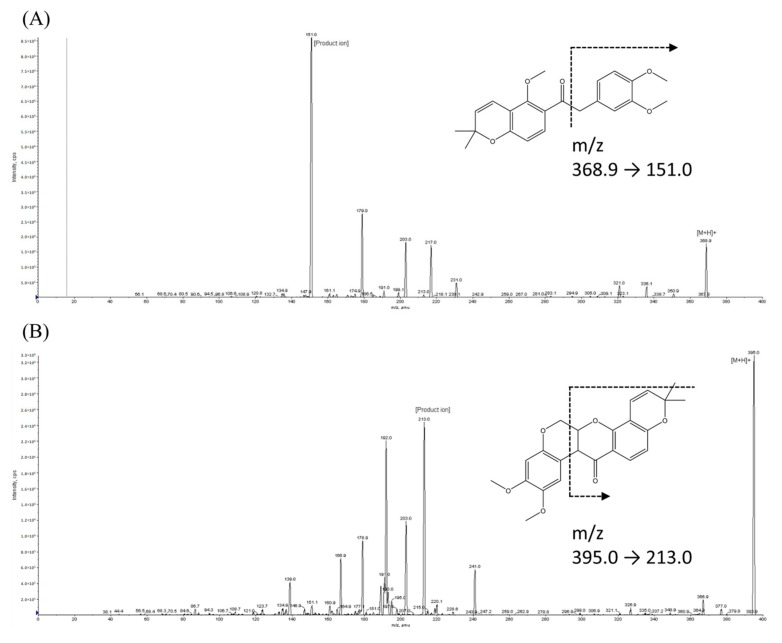
The product-ion scan spectra and proposed multiple reaction monitoring (MRM) transitions of (**A**) SH-1242 and (**B**) deguelin, (the internal standard).

Based on the chemical structures and product-ion spectra of SH-1242 and internal standard (IS) (Figure 1), *m*/*z* transition values were set at 368.9→151.0 for SH-1242 and 395.0→213.0 for the IS (deguelin). For the IS, this *m*/*z* transition value was comparable to the fragmentation pattern found in the literature [12,17]. Isocratic flow with the run time of 3 min per sample resulted in adequate chromatographic separations for SH-1242 and IS without any apparent interfering peaks (Supplementary Figure S1). SH-1242 and the IS were adequately resolved with the retention times of 1 min for SH-1242 and 0.96 min for IS. These observations indicated that the analytical method in this study allowed adequate throughput for the chromatographic separation of SH-1242 with a reasonable resolution. Therefore, the chromatographic conditions were used for subsequent analyses.

### 2.2. Selectivity

Representative ion chromatograms of double blanks, zero blanks, and lower limit of quantification (LLOQ) samples are shown in Supplementary Figure S1. The results obtained from six replicates of double blank, zero blank, and LLOQ samples showed that no appreciable interfering peak was evident in the vicinity of the retention times for the analyte and IS peaks (Table 1). At the LLOQ level (1 ng/mL for rat plasma and 2 ng/mL for mouse plasma), the precision of the peak area was found to be 5.15% and 10.5% for rat and mouse plasma, respectively. Taken together, these observations showed that the current HPLC-MS/MS assay provided adequate selectivity for the analysis of SH-1242 in rat and mouse plasma samples.

### 2.3. LLOQ and Linearity

The concentration levels of SH-1242 at which the signal-to-noise ratio was consistently 10 or more with acceptable precision (i.e., less than 20% CV) was found to be 1 ng/mL for rat plasma and 2 ng/mL for mouse plasma. Calibration curves (Supplementary Figure S2), including the LLOQ, were apparently linear over the specified concentration ranges for rat and mouse plasma (Table 2). Consistent with this statement, the correlation coefficients (i.e., R values with the weighting factor of 1/x^2^) of the calibration curves ranged from 0.995 to 0.999, with all the calibrators found to be within the acceptance criterion of 15% (20% at LLOQ).

### 2.4. Accuracy, Precision, and Dilution Integrity

The accuracies and precisions of QC samples of SH-1242 in rat and mouse plasma are summarized in Table 3. Intra-day (inter-day) precisions of QC samples ranged from 3.85% to 12.7% (2.84% to 12.8%) and from 2.55% to 3.09% (3.57% to 6.72%) for the rat and mouse plasma, respectively; the intra-day (inter-day) accuracies (as a relative error, RE%) of QC samples ranged from −5.15% to 4.83% (−2.83% to 4.63%) in rat plasma and –6.02% to 1.42% (−2.53% to 1.63%) in mouse plasma. In addition, when SH-1242 samples of concentration of 8000 ng/mL (i.e., exceeding the ULOQ of 1000 ng/mL) were 10-fold diluted, calculated mean intra-day (inter-day) concentrations were 764 ng/mL (743 ng/mL) with a CV value of 1.39% (2.84%) for rats and 757 ng/mL (782 ng/mL) with a CV value of 4.47% (6.72%) for mice, indicating that plasma samples with SH-1242 concentrations exceeding ULOQ could be diluted for analysis. Taken together, these results indicated that the current assay was accurate and precise for the estimation of SH-1242 concentration in plasma samples obtained from rats or mice.

### 2.5. Matrix Effect, Extraction Efficiency, and Recovery

Matrix effect, recovery, and extraction efficiency for SH-1242 in rat and mouse plasma samples are summarized (Table 4). The mean extraction efficiencies ranged from 107% to 119% for rat plasma and from 91.1% to 107% for mouse plasma, indicating that the loss of the analyte through the extraction process was not significant in both matrices. However, the matrix effect of SH-1242 ranged from 82.2% to 92.8% for rat plasma and from 44.7% to 48.0% for mouse plasma. For rats, the recovery (or IS-normalized recovery) of SH-1242 ranged from 91.3% to 108% (103% to 118%). In line with this, the recovery of other rotenoids from human serum (e.g., rotenone, rotenolone, and deguelin) was reported to be in a range from 92.3% to 115% [17]. In contrast, the recovery of SH-1242 after the extraction from mouse plasma was ranged from 43.7% to 47.8% (Table 4). Collectively, these observations indicated that there were distinct differences in the matrix effects and IS-normalized recoveries of rotenoid compounds in biological matrices between human/rat and mouse. These discrepancies might be related to the different SH-1242 LLOQ values observed for the two matrices (i.e., 1 ng/mL for rat plasma vs. 2 ng/mL for mouse plasma): It is possible that factors influencing the detection process of the rotenoids (e.g., electrospray ionization in HPLC-MS/MS interface) [18,19] are different between the matrices. Nevertheless, variabilities in peak responses used for the calculation of the recovery parameters were consistently less than 15% (Table 4) for rat and mouse plasma. In addition, no appreciable difference was found on essential assay parameters for the two matrices (e.g., accuracies and precisions). Therefore, despite the differences in the matrix effect/recovery in both biological matrices, we assumed that the devised assay was still applicable for pharmacokinetic studies involving the animal species and the applicability subsequently tested.

### 2.6. Stability

The stability of SH-1242 and IS in stock solutions was studied at concentrations of 1 mg/mL and 500 ng/mL, respectively, under different storage conditions. As summarized in Table 5, the relative responses of SH-1242 and IS stock solutions compared to those at time zero (i.e., the reference value) were ranged from 85.2% to 109% and from 86.2% to 105%, respectively. In addition, when QC samples, containing SH-1242 at three different concentration levels for rat or mouse plasma, were subjected to various handling and storage conditions (Table 6), we found that benchtop stability at room temperature for 24 h, autosampler stability at 4 °C for 3 days, stability after three freeze-thaw cycles, and long term stability at 4 °C for 2 weeks were all acceptable with CV and RE values ranging from 0.825% to 8.54% and −8.71% to 4.92% for rat plasma, and from 0.699% to 12% and −4.08% to 12% for mouse plasma, respectively. Collectively, these observations suggested that SH-1242 and deguelin were adequately stable in rat and mouse plasma under various handling and storage conditions.

### 2.7. Applicability of the Assay to Pharmacokinetic Studies

**Figure 2:**
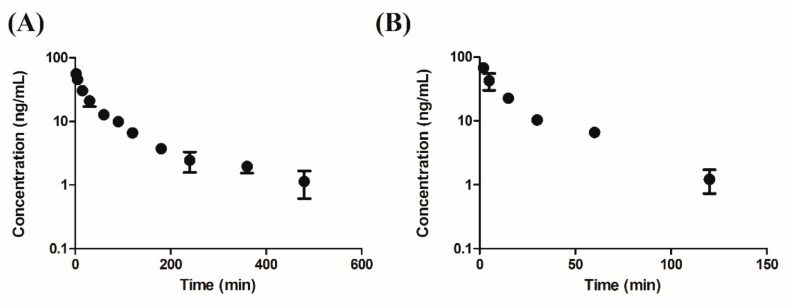
Mean concentration-time profiles of SH-1242 after (**A**) an intravenous injection of 0.1 mg/kg SH-1242 (*n* = 4) to rats and (**B**) an intravenous injection of 0.1 mg/kg SH-1242 (*n* = 3 for each time point) to mice. Results are presented as means ± standard deviations.

To determine whether the current assay could be applied to pharmacokinetic studies on SH-1242 in preclinical animal species, SH-1242 was intravenously administered to rats or mice at a dose of 0.1 mg/kg. Concentration-time profiles of SH-1242 in rats and mice are shown in Figure 2, and calculated pharmacokinetic parameters, including T_1/2_, CL, area under the curve from time zero to infinity (AUC_inf_), mean residence time (MRT), and V_ss_, are listed in Table 7. For rats, AUC_inf_, CL, V_ss_, and T_1/2_ values of SH-1242 were 3350 ± 573 ng·min/mL, 30.5 ± 4.49 mL/min/kg, 4380 ± 716 mL/kg, and 146 ± 59 min, respectively. Interestingly, SH-1242 appeared to have lower systemic clearance and relatively limited distribution space, compared to those of deguelin, a model rotenoid, in rats (e.g., CL value of 72.7 mL/min/kg, and V_ss_ value of 30.5 L/kg) [20]. In mice, AUC_inf_, CL, V_ss_, and T_1/2_ values of SH-1242 were 1440 ng·min/mL, 69.4 mL/min/kg, 2060 mL/kg, and 26.3 min, respectively. In general, SH-1242 concentrations were readily measurable in all plasma samples in both animals at up to 8 h after the intravenous administration, indicating that the developed method could be readily applicable for the characterization of the pharmacokinetics of SH-1242 in preclinical study settings.

## 3. Materials and Methods

### 3.1. Chemicals, Reagents, and Experimental Animals

SH-1242 (purity of >99%) and deguelin (purity of >99%, IS of this study) were synthesized, as previously described [9]. Methanol and acetonitrile (high-performance liquid chromatography (HPLC) grade) were purchased from Fisher Scientific (Pittsburgh, PA, USA). Double-distilled water (DDW) was prepared in-house using a Millipore Simplicity water purification system (Millipore, Bedford, MA, USA). Formic acid (FA) was purchased from Sigma-Aldrich (St. Louis, MO, USA). Blank rat and mouse plasma samples were collected from animals provided by Orient Bio Inc. (Gyeonggi-do, Korea). Experimental protocols involving animals used in this study were carefully reviewed by the Seoul National University Institutional Animal Care and Use Committee (IACUC), in accordance with the ‘Principles of Laboratory Animal Care’ guideline, published by the National Institutes of Health publication number 85-23, revised 1985 (SNU-170303-1 and SNU-170120-4-4).

### 3.2. HPLC Conditions

A Waters e2695 HPLC system (Milford, MA, USA) consisting of a binary pump, an online degasser, an autosampler, a column heater, and a reversed-phase HPLC column (Poroshell 120 EC-C18 2.7 µm (4.6 mm × 50 mm, Agilent, Santa Clara, CA, USA)) was used for chromatographic separations. The mobile phase was composed of 0.1% FA in acetonitrile and 0.1% FA in DDW in a ratio of 80:20 and was isocratically delivered at a flow rate of 1 mL/min. Throughout the assay, the temperatures of analytical samples and the column were maintained at 4 °C and 25 °C, respectively, and the run time was 3 min for each sample.

### 3.3. Mass Spectrometer Conditions

In this study, mass spectrometric detection was conducted using API 3200 Qtrap^®^ (Applied Biosystems, Foster City, CA, USA), equipped with an electrospray ionization (ESI) source in the positive ion mode. Multiple reaction monitoring (MRM) method was used to quantify the analytes: m/z transitions were monitored at 368.9→151.0 for SH-1242 and 395.0→213.0 for IS. After a series of optimization studies to secure a sensitive and robust response of the signal, conditions, such as ion spray voltage, source temperature, and the three source gas pressure values (i.e., curtain gas pressure, ion source gas 1 and 2), were determined to be 5000 V, 200 °C, and 50 psi, respectively (Supplementary Figure S3). In addition, using the quantitative optimization mode built-in Analyst^TM^ software (version 1.4.2, Applied Biosystems, Waltham, MA, USA), the following factors were obtained: Declustering potentials for SH-1242 and IS were 41 V and 61 V, respectively, and entrance potentials were 4.5 V and 7 V, collision energies were 27 V and 29 V, and the collision cell exit potential was 4 V for both analytes. In this study, the Analyst^TM^ software was used for data acquisition and quantification.

### 3.4. Standards and Quality Control (QC) Samples

Stock solutions of SH-1242 and IS were prepared at the concentrations of 1 mg/mL and 500 ng/mL in methanol, respectively. A serial dilution of the SH-1242 stock solution with methanol was carried out to obtain a set of SH-1242 standard solutions, and 5 µL aliquots of these standard solutions were added to 45 µL of blank plasma to prepare SH-1242 calibration standards at concentrations of 1, 2, 5, 10, 20, 50, 100, 200, 500, and 1000 ng/mL for rat plasma and 2, 5, 10, 20, 50, 100, 200, 500, and 1000 ng/mL for mouse plasma. Using similar dilution protocol, a batch of QC samples for SH-1242 was prepared at concentrations of 1 (LLOQ), 2 (low QC), 40 (mid QC), and 800 ng/mL (high QC) for rat plasma and 2 (LLOQ), 4 (low QC), 40 (mid QC), and 800 ng/mL (high QC) for mouse plasma. Samples were then processed according to the procedure described in Section 3.5.

### 3.5. Sample Preparation

A total of 50 µL aliquots of plasma samples, calibration standards, or QC samples were transferred to Safeseal Microcentrifuge Tubes (Sorenson BioScience, Murray, UT, USA), followed by the addition of 200 µL of IS stock solution. Mixtures were vortex-mixed for 5 min and centrifuged at 16,100 g for 5 min at 4 °C. Supernatants were subsequently transferred to fresh analysis vials (MicroSolv Technology Corporation, Leland, NC, USA), and 50 µL aliquots were injected onto the HPLC-MS/MS system. In this study, the injection volume (i.e., 50 µL) was less than 10% of the void volume of the column of 830 µL (i.e., 4.6 mm of inner diameter and 50 mm of length) and did not appear to have any appreciable impact on the performance of the instrument (e.g., analyte peak width, retention time, and carryover) [21].

### 3.6. Method Validation

#### 3.6.1. Selectivity

Six lots of pooled rat and mouse plasma samples were used to evaluate assay selectivity. The presence of any interfering peak in double blank samples (i.e., blank plasma without SH-1242 or IS), zero blank samples (i.e., blank plasma with IS only), and LLOQ samples was carefully monitored. In this study, assay selectivity was assumed adequate when no apparent interfering peaks were observed in the vicinities of the analyte peaks.

#### 3.6.2. LLOQ and Linearity

The LLOQ of SH-1242 was determined to be the minimum concentration, with a signal-to-noise ratio being consistently greater than 10. Throughout our preliminary studies, LLOQ values of SH-1242 in the rat and mouse plasma were determined to be 1 ng/mL and 2 ng/mL, respectively, and these concentrations were subsequently used in the development of the assay. Various concentrations of SH-1242, ranging from 1 ng/mL to 1000 ng/mL for rat plasma (ten concentration levels) and 2 ng/mL to 1000 ng/mL for mouse plasma (nine concentration levels), were used to determine the linearity of the assay. A calibration curve was constructed by plotting SH-1242-to-IS peak area ratios against nominal SH-1242 concentrations. The linear least-square regression method with a weighting factor of 1/x^2^ was used to determine the slope and y-intercept.

#### 3.6.3. Precision, Accuracy, and Dilution Integrity

Accuracy and precision within and between runs were assessed using five separate batches of QC samples. Each batch consisted of six replicates of QC samples at four concentration levels (LLOQ, 2 or 4 ng/mL, 40 ng/mL, and 800 ng/mL, in rat or mouse plasma, respectively). The accuracy of the assay was determined by calculating the percent differences between the calculated and theoretical concentrations, and the precision of the method was defined as the coefficient of variation (CV) percentage at each concentration. The method was considered accurate when the calculated concentrations of QC samples were within 15% of nominal concentrations. In addition, the assay was considered precise when the CV of calculated concentrations of QC samples was 15% or less (viz, 20% CV for LLOQ samples).

When it was necessary to study dilution integrity of the assay, a fresh batch of six-replicate samples (50 µL) was first prepared in rat or mouse plasma at an SH-1242 concentration of 8000 ng/mL (i.e., at a concentration exceeding the upper limit of quantification (ULOQ)). Samples were then diluted 10-fold with blank plasma (with 450 µL) to have the expected plasma concentration of 800 ng/mL and to bring the final concentration within the calibration range. Diluted samples were then processed and analyzed, as described in Section 3.5.

#### 3.6.4. Matrix Effect, Extraction Efficiency, and Recovery

The extent of matrix effect, extraction efficiency, and recovery of SH-1242 in the rat and mouse plasma was evaluated by analyzing three sets of plasma standards at three different concentration levels (2 ng/mL, 40 ng/mL, and 800 ng/mL) [22,23]. Pre-spiked extracted samples (Set 1; extracted after the addition of analyte to blank plasma) were prepared according to the procedure described in Section 3.5. Similarly, post-spiked extracted samples were prepared by processing blank plasma and then adding SH-1242 to have the prescribed concentrations. Extraction efficiency was determined by dividing the mean peak areas of pre-spiked extracted samples (Set 1) by those of post-spiked extracted samples. Mean peak areas of the pre- and post-spiked extraction samples were divided by the mean peak areas of neat standard solutions of the analyte in methanol containing 0.1% FA (Set 2) to determine the recovery and matrix effect, respectively.

#### 3.6.5. Stability

The stability of SH-1242 and IS was evaluated under various storage and handling conditions. Stock solutions of SH-1242 and IS at concentrations of 1 mg/mL and 500 ng/mL, respectively, were stored at room temperature (25 °C) for 6 h, under refrigerated conditions (4 °C) for 24 h or 2 weeks, or under two different frozen conditions (−20 °C and −80 °C) for 2 weeks. Solutions of SH-1242 were diluted to 50 ng/mL prior to analysis. In addition, QC samples at three different concentrations (2 or 4 ng/mL, 40 ng/mL, and 800 ng/mL) were prepared and processed. The samples were then placed in various handling conditions (i.e., standing in an autosampler (4 °C for 3 days) or under bench-top condition (25 °C for 24 h), or subjected to three freeze-thaw cycles, or long-term refrigeration (4 °C for 2 weeks)). Mean peak areas of stock solutions were compared with those of fresh stock solutions. For QC samples, the results were compared with nominal concentrations. In this study, analytes were considered stable under test conditions when accuracies at each concentration were within 15% of nominal concentration.

### 3.7. Application of the Assay to Pharmacokinetic Studies of SH-1242

To test the applicability of the current assay to pharmacokinetic studies, in preclinical animal species, male Sprague-Dawley rats (weighing 250–270 g) or ICR mice (weighing 18–20 g) were used. Prior to the experiment, animals fasted for 12 h with free access to water. SH-1242 was dissolved in a dosing vehicle consisting of dimethyl sulfoxide, PEG400 (Sigma-Aldrich, St. Louis, MO, USA), and normal saline in a ratio of 1:6:3 (*v*/*v*/*v*%), and intravenously bolus-injected to the right femoral vein at 2 mL/kg for rats or to the tail vein at 5 mL/kg for mice. In this study, the intravenous dose of SH-1242 was set at 0.1 mg/kg for both species. Blood samples (approximately 150 µL each) were collected in heparinized tubes via the right femoral artery at 2, 5, 15, 30, 60, 90, 120, 180, 240, 360, and 480 min after the administration to rats (*n* = 4) and via the retro-orbital plexus at 2, 5, 15, 30, 60, or 120 min after the administration to mice (*n* = 3 for each time point). For the mouse study, a blood sample was obtained once from each animal, and the animal was sacrificed after collection. Plasma was obtained by centrifuging blood samples at 16,100 *g* for 5 min at 4 °C and then processed, as described in Section 3.5. The plasma concentration versus time data was analyzed using the non-compartmental method in the WinNonlin software (WinNonlin Professional 5.0.1.; Pharsight, Mountain View, CA, USA) to calculate essential kinetic parameters, such as terminal phase half-life (T_1/2_), systemic clearance (CL), and steady-state volume of distribution (V_ss_).

## 4. Conclusions

A straightforward and rapid HPLC-MS/MS assay was developed and validated for the quantification of SH-1242 in rat and mouse plasma. The method was validated in terms of its selectivity, linearity, accuracy, precision, dilution, matrix effects, recovery, and stability. Assay parameters were found to comply with the acceptance criteria described in U.S. FDA guidelines. The developed assay was found to be suitable for pharmacokinetic studies on SH-1242, involving rats and mice.

## Figures and Tables

**Table 1:** The specificity of SH-1242 in rat and mouse plasma.

MATRIX LOT	Response (Peak Area)							
	Rat Plasma				Mouse Plasma			
	Double Blank ^a^	Zero Blank ^b^	LLOQ (1 ng/mL)	HQC (800 ng/mL)	Double Blank	Zero Blank	LLOQ (2 ng/mL)	HQC (800 ng/mL)
1	0	0	209	71,500	0	0	150	23,800
2	0	0	214	80,700	0	0	137	24,500
3	0	0	212	77,100	0	0	121	22,100
4	0	0	225	78,000	0	0	119	22,400
5	0	0	238	77,600	0	0	114	23,700
6	0	0	211	77,200	0	0	134	24,400
Mean	0	0	218	77,000	0	0	129	23,500
CV(%) ^c^	0	0	5.15	3.91	0	0	10.5	4.30

^a^ Double blank: sample containing no analyte or IS. ^b^ Zero blank: sample containing only IS. ^c^ Coefficient of variation (CV%) = (standard deviation/mean) × 100. IS: internal standard, LLOQ: lower limit of quantification, HQC: high QC.

**Table 2:** Calibration curves for SH-1242 in rat and mouse plasma.

Run	Rat Plasma			Mouse Plasma		
	Slope	Intercept	R	Slope	Intercept	R
1	0.00349	0.00340	0.999	0.00633	0.00982	0.995
2	0.00329	0.00281	0.999	0.00450	0.00679	0.999
3	0.00339	0.00227	0.998	0.00515	0.00378	0.999
4	0.00335	0.00402	0.999	0.00583	0.00897	0.996
5	0.00337	0.00417	0.999	0.00616	0.00194	0.998
Mean	0.00338	0.00333	0.999	0.00559	0.00626	0.997
CV(%) ^a^	2.16	-	-	13.6	-	-

^a^ Coefficient of variation (CV%) = (standard deviation/mean) × 100.

**Table 3:** Accuracy and precision of SH-1242 determinations in rat and mouse quality control (QC) samples.

Batch	Rat Plasma					Mouse Plasma				
	Theoretical Concentration (ng/mL)					Theoretical Concentration (ng/mL)				
	LLOQ	LQC	MQC	HQC	HQC ^a^	LLOQ	LQC	MQC	HQC	HQC ^a^
	1	2	40	800	800	2	4	40	800	800
Intra-day (*n* = 6)										
Mean	1	1.98	41.9	759	764	1.95	4.06	39.7	752	757
Precision (CV%) ^b^	12.7	4.64	4.41	3.85	1.39	2.56	2.55	2.96	3.09	4.47
Accuracy (RE%) ^c^	0.38	−0.83	4.83	−5.15	−4.48	−2.42	1.42	−0.83	−6.02	−5.35
Inter-day (*n* = 30)										
Mean	1.04	2.04	41.9	777	743	1.99	4.04	40.7	780	782
Precision (CV%) ^b^	12.8	5.04	5.03	4.24	2.84	5.87	4.43	3.57	5.27	6.72
Accuracy (RE%) ^c^	3.95	2.22	4.63	−2.83	−7.15	−0.73	0.91	1.63	−2.53	−2.30

^a^ Analyzed after a ten-fold dilution with blank plasma. ^b^ CV(%) = (standard deviation/mean) × 100. ^c^ RE(%) = [(calculated concentration – theoretical concentration)/theoretical concentration] × 100. RE: relative error, LQC and MQC: low and mid QC.

**Table 4:** Matrix effect, extraction efficiency, and recovery of the assay for SH-1242 determination in rat and mouse plasma samples.

Nominal Concentration (ng/mL)	Matrix Effect (%) ^a^	Extraction Efficiency (%) ^b^	Recovery (%) ^c^	IS-Normalized Recovery (%) ^c^	CV (%) ^d^			
					Analyte		IS	
Rat plasma					Set 1	Set 2	Set 1	Set 2
2	92.8	107	99	110	9.08	2.92	5.95	2.13
40	90.4	119	108	118	7.11	6.05	2.96	2.01
800	82.2	111	91.3	103	2.31	6.72	3.12	1.95
Mouse plasma					Set 1	Set 2	Set 1	Set 2
2	45.9	101	46.3	74.4	6.38	2.92	6.77	2.13
40	44.7	107	47.8	74.3	7.10	6.05	2.65	2.01
800	48	91.1	43.7	73.4	10.5	6.72	7.52	1.95

^a^ Matrix effect was calculated by expressing the ratio of the mean peak area of the analyte added after extraction to the mean peak area of neat standard solution (Set 2) of the analyte multiplied by 100. ^b^ Extraction efficiency was calculated by dividing the mean peak area of the analyte added before extraction (Set 1) by the mean peak area of the analyte added after extraction multiplied by 100. ^c^ Recovery (IS-normalized recovery) was calculated by the ratio of the mean peak area of the analyte (normalized by IS peak area) added before extraction (Set 1) to the mean peak area of a neat standard solution of the analyte (normalized by IS peak area) (Set 2) multiplied by 100. ^d^ CV was calculated as a standard deviation of the peak area divided by the mean peak area multiplied by 100.

**Table 5:** Stability of SH-1242 and IS in stock solutions under typical storage conditions.

Batch (*n* = 3)	Response (Peak Area)					
	Initial (0 h)	Room Temp. (6 h)	Refrigerated (4 °C, 24 h)	Refrigerated (4 °C, 2 Weeks)	Refrigerated (−20 °C, 2 Weeks)	Refrigerated (−80 °C, 2 Weeks)
SH-1242 ^a^						
Mean	18,000	19,700	19,000	15,400	16,100	16,400
CV(%) ^b^	3.93	13.5	11.1	9.14	5.44	3.01
Relative conc. (%) ^c^	100	109	105	85.2	89.1	90.8
IS						
Mean	19,100	19,200	20,000	17,600	17,400	16,400
CV(%)	4.27	6.20	0.762	4.65	8.36	1.53
Relative conc. (%)	100	101	105	92.3	91.4	86.2

^a^ Stock solutions of SH-1242 were diluted to 50 ng/mL prior to analysis. ^b^ CV(%) = (standard deviation/mean) × 100. ^c^ Relative concentrations (%) were obtained by dividing measured values by initial values. IS: internal standard

**Table 6:** Stability of SH-1242 QC samples under typical storage conditions.

Batch	Rat Plasma			Mouse Plasma		
	Theoretical Concentration (ng/mL)			Theoretical Concentration (ng/mL)		
	LQC	MQC	HQC	LQC	MQC	HQC
	2	40	800	4	40	800
Benchtop stability at room temperature (25 °C) for 24 h (*n* = 3)						
Mean	1.87	37.3	737	4.21	41.7	810
Precision (CV%) ^a^	6.95	4.35	2.62	9.47	4.38	5.57
Accuracy (RE%) ^b^	–6.33	–6.83	–7.88	5.25	4.25	1.25
Autosampler stability at 4 °C for 3 days (*n* = 3)						
Mean	2.00	38.9	773	4.19	42.6	863
Precision (CV%)	8.27	6.05	3.16	4.90	5.15	0.699
Accuracy (RE%)	–0.167	–2.75	–3.33	4.67	6.50	7.83
Freeze-thaw stability (3 cycles, *n* = 3)						
Mean	1.84	41.97	780	4.45	42.4	840
Precision (CV%)	3.85	4.20	3.57	2.00	4.32	1.33
Accuracy (RE%)	–7.83	4.92	–2.50	11.3	6.00	5.00
Long term stability at 4 °C for 2 weeks (*n* = 3)						
Mean	2.02	37.3	730	3.84	44.8	865
Precision (CV%)	8.54	2.56	0.825	12.0	1.77	4.37
Accuracy (RE%)	0.833	–6.75	–8.71	–4.08	12.0	8.08

^a^ CV(%) = (standard deviation/mean) × 100. ^b^ RE(%) = [(calculated concentration – theoretical concentration)/theoretical concentration] × 100.

**Table 7:** Pharmacokinetic parameters of SH-1242 after a single intravenous administration at a dose of 0.1 mg/kg to rats and mice.

Pharmacokinetic Parameters (Units)	Rats	Mice
	Mean ± S.D.	Representative ^a^
T_1/2_ (min)	146 ± 59	26.3
CL (mL/min/kg)	30.5 ± 4.49	69.4
AUC_inf_ (ng·min/mL)	3350 ± 573	1440
MRT (min)	149 ± 46.9	29.6
V_ss_ (mL/kg)	4380 ± 716	2060

^a^ Because of the study design (i.e., one-time point sample per mouse), the calculation of the standard deviation was not possible for pharmacokinetic parameters in mice.

## Supplementary Materials

The following are available online. Figure S1: Multiple reaction monitoring (MRM) chromatograms of (A) double blank rat plasma, (B) zero blank rat plasma containing 500 ng/mL IS, (C) rat plasma containing SH-1242 at LLOQ (1 ng/mL) and IS, (D) double blank mouse plasma, (E) zero blank mouse plasma containing 500 ng/mL IS, and (F) mouse plasma containing SH-1242 at LLOQ (2 ng/mL) and IS. Figure S2: Calibration curves for the LC-MS/MS analysis of SH-1242 in (A) the rat and (B) mouse plasma (i.e., 5 separate runs). Data are presented as means ± standard deviations. Slopes and intercepts of linear regression lines are showed in Table 2. Figure S3: Dependency of variables related to source/gas on the signal intensity in turbo VTM ion spray ionization mode. Closed circles and error bars represent the mean and upper/lower response of the signal during 1 min infusion of SH-1242 solution at 1 μg/mL (in 50% methanolic solution containing 0.1% FA).
