# Supplementary material for: Development and Validation of Analytical Method for SH-1242 in the Rat and Mouse Plasma by Liquid Chromatography/Tandem Mass Spectrometry
Source: Molecules. 2020 Jan 25;25(3):531. doi: 10.3390/molecules25030531 (PMC7037321; doi:10.3390/molecules25030531)
Supplement: Supplementary file 1 [file molecules-25-00531-s001.pdf]

Supplementary Materials

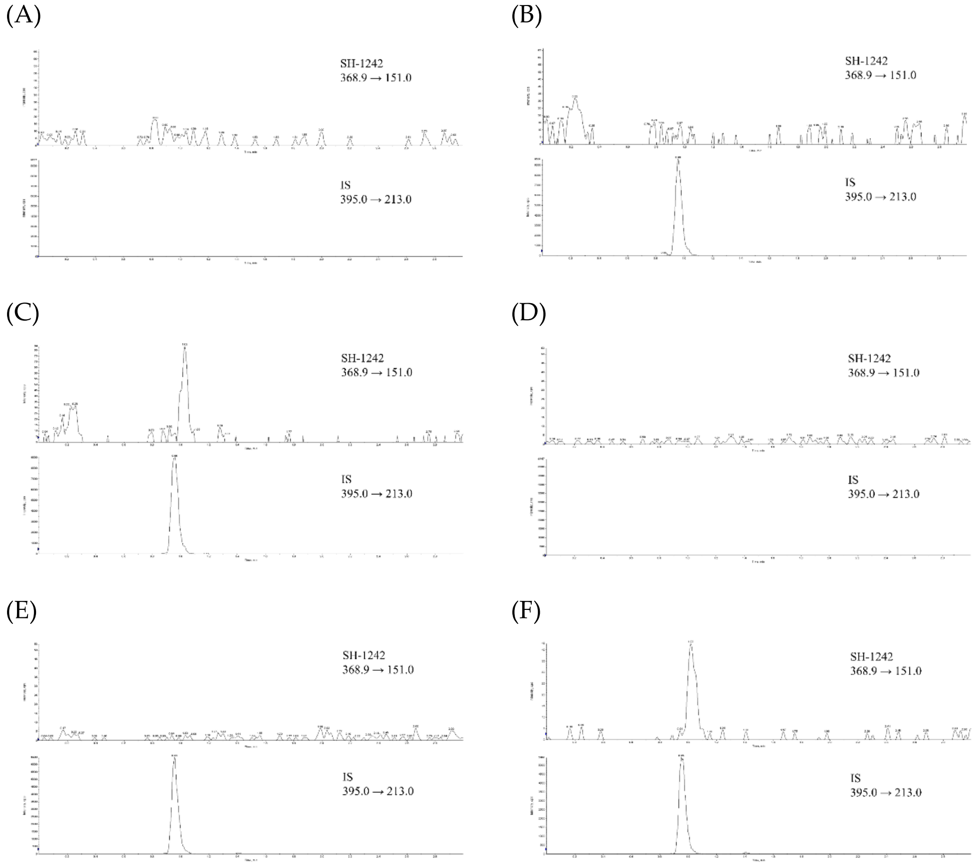

Figure S1. Multiple reaction monitoring (MRM) chromatograms of (A) double blank rat plasma, (B) zero blank rat plasma containing 500 ng/mL IS, (C) rat plasma containing SH-1242 at LLOQ (1 ng/mL) and IS, (D) double blank mouse plasma, (E) zero blank mouse plasma containing 500 ng/mL IS, and (F) mouse plasma containing SH-1242 at LLOQ (2 ng/mL) and IS.

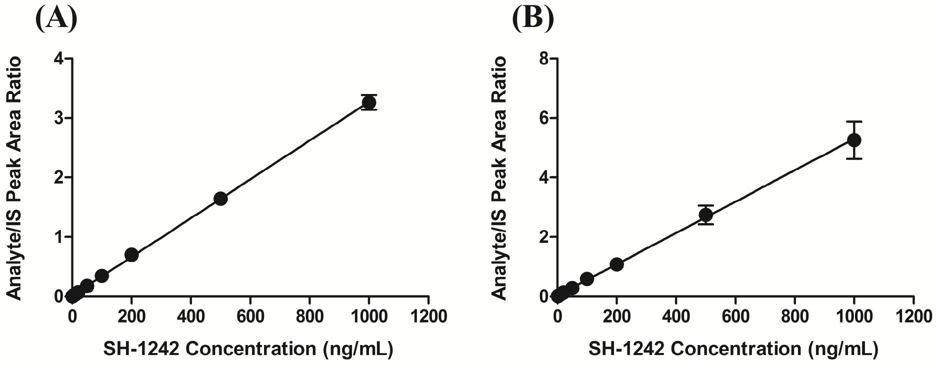

Figure S2. Calibration curves for the LC-MS/MS analysis of SH-1242 in (A) the rat and (B) mouse plasma (i.e., 5 separate runs). Data are presented as means ± standard deviations. Slopes and intercepts of linear regression lines are showed in Table 2.

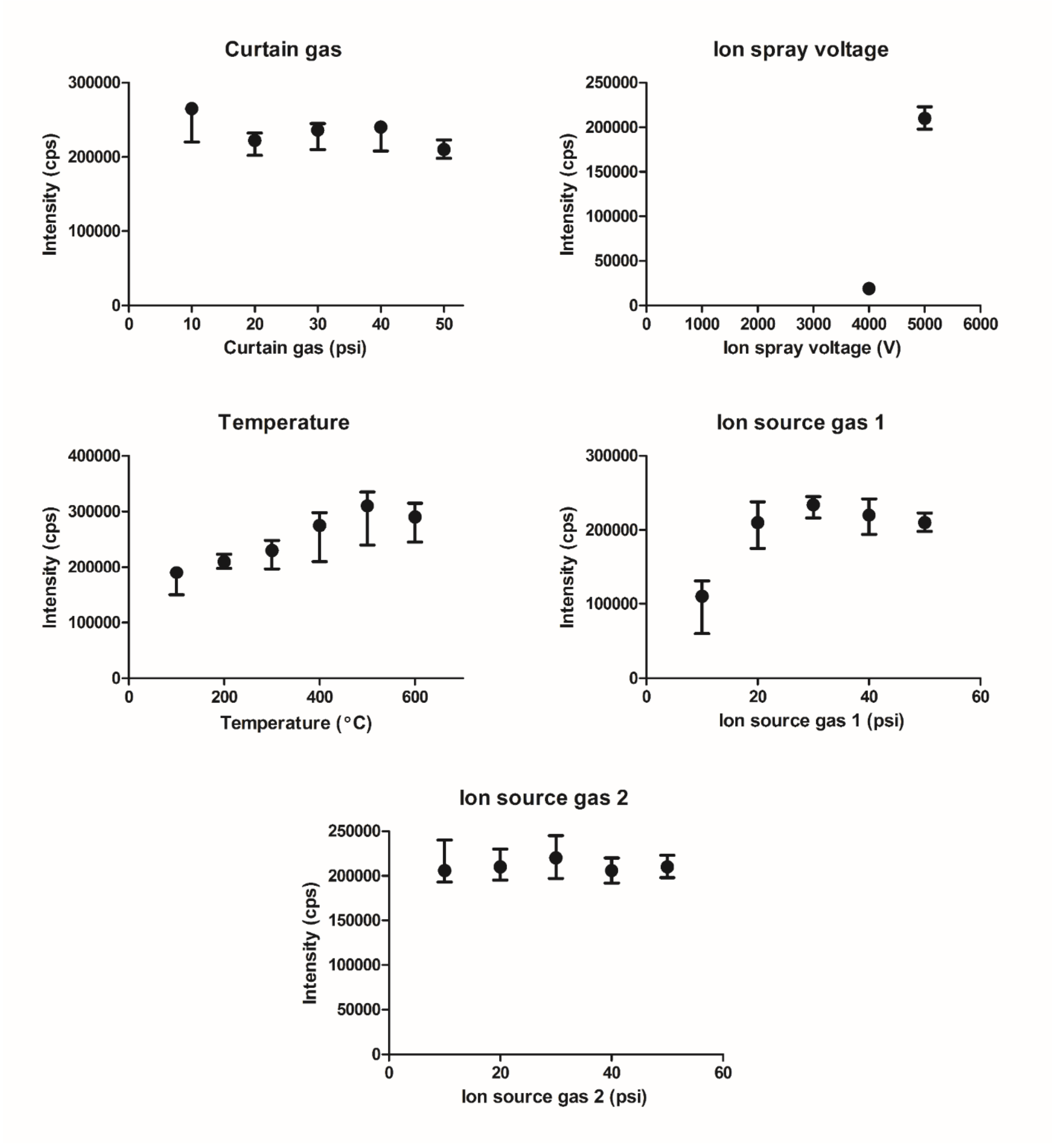

Figure S3. Dependency of variables related to source/gas on the signal intensity in turbo VTM ion spray ionization mode. Closed circles and error bars represent the mean and upper/lower response of the signal during 1 min infusion of SH-1242 solution at 1 µg/mL (in 50% methanolic solution containing 0.1% FA)
